# Educational supervision and the impact of workplace-based assessments: a survey of psychiatry trainees and their supervisors

**DOI:** 10.1186/1472-6920-9-51

**Published:** 2009-07-29

**Authors:** T Everett Julyan

**Affiliations:** 1 Clinic K, Crosshouse Hospital, Ayrshire KA2 0BE, UK

## Abstract

**Background:**

Educational supervision (ES) is considered to be an essential component of basic specialist training in psychiatry in the UK. However, previous studies have indicated variation in its provision, and uncertainty about structure and content. Workplace-based assessments (WPBAs) were introduced in 2007 as part of major postgraduate medical training reform. Placing considerable time demands on trainees and supervisors alike, the extent to which WPBAs should utilise ES time has not been specified. As ES and WPBAs have discrete (although complementary) functions, there is the potential for this increased emphasis on assessment to displace other educational needs.

**Methods:**

All junior doctors and their educational supervisors in one UK psychiatry training scheme were surveyed both before and after the introduction of WPBAs. Frequency and duration of ES were established, and structure, content and process were ascertained. Opinions on usefulness and responsibility were sought. The usage of ES for WPBAs was also assessed.

**Results:**

The response rate of 70% showed general agreement between trainees and supervisors, but some significant discrepancies. Around 60% reported 1 hour of ES taking place weekly or 3 times per month. Most agreed that responsibility for ES should be shared equally between trainees and supervisors, and ES was largely seen as useful. Around 50% of trainees and supervisors used 25–50% of ES time for WPBAs, and this did not appear to affect the usefulness of ES or the range of issues covered.

**Conclusion:**

ES continues to be an important component of psychiatric training. However, using ES for WPBAs introduces the potential for tension between trainees' education and their assessment by emphasising certain training issues at the expense of others. The impact of reduced training time, WPBAs and uncertainties over ES structure and content should be monitored to ensure that its benefits are maximised by remaining tailored to individual trainees' needs.

## Background

In the UK, the Royal College of Psychiatrists (RCPsych) has long considered educational supervision (ES) to be an essential component of basic specialist training in psychiatry, and has published detailed guidance for both trainees and their supervisors [1,2]. The purpose of ES is specifically educational rather than clinical, including both practical skills and theoretical learning, and must be provided for 1 hour weekly by a designated educational supervisor (usually the trainee's consultant). Moreover, this time belongs exclusively to the trainee, with a focus on their personal development and learning needs, although appraisal and assessment are also integral to the process [3]. Until recently, trainees were expected to use the RCPsych Logbook to record relevant information and to monitor progress [4].

Previous studies have reported generally positive views of ES among both trainees and supervisors [5-9]. However, several issues have been highlighted, including variation in its provision, uncertainty over its purpose, structure and content, and a desire for more detailed guidelines and training for supervisors. There is, therefore, a need to continuously monitor and improve this essential part of postgraduate training, especially in light of recent developments in the assessment of junior doctors in the UK.

Workplace-based assessments (WPBAs) were introduced in August 2007 as part of major reforms in postgraduate medical training in the UK and the RCPsych curriculum [2,10]. Their purpose is assessment, facilitating multidimensional, broad evaluation of a doctor's longer-term performance rather than the traditional, limited, one-off assessments traditionally associated with formal examinations [11]. The satisfactory completion of a specified number of a range of WPBAs is mandatory before trainees can progress to the next stage. WPBAs are based on laudable principles, but they place additional time demands on trainees and supervisors alike. This is particularly challenging in the present UK climate due to the ongoing reduction in working hours available for training, associated with changing shift patterns and the European Working Time Directive (which restricts junior doctors to 48 hours of work weekly by August 2009, with mandatory limits to shift length and requirements for regular rest periods) [12-14].

At least partly because of time constraints, educational supervisors expect to use ES time for WPBAs. There is the potential for ES to be enhanced by this, e.g. by increasing trainees' motivation and providing formative content [11]. However, while ES and WPBAs have discrete (although complementary) functions, the RCPsych has published no specific guidance on the extent to which existing ES time should be used for WPBAs. As noted above, previous studies have reported significant variation in the provision of ES, and it appears that not all trainees receive even the minimum ES required by the RCPsych. With the increased emphasis on assessment via WPBAs but less time available for current responsibilities, ES may therefore become more of a priority for trainees and supervisors alike. Conversely, there is the potential for existing educational needs usually addressed within ES to be displaced by WPBAs.

This study aims to address two key questions. Firstly, how does the current provision of ES in a typical UK basic psychiatry training scheme compare with the RCPsych requirements? And secondly, does using ES for WPBAs affect its educational usefulness?

## Methods

Junior doctors in the NHS Ayrshire & Arran basic psychiatry training scheme were surveyed both before and after the introduction of WPBAs. This permitted assessment of the frequency, duration, structure and content of ES, as well as any impact of WPBAs, in a UK psychiatry training scheme of average size (15 trainees). After an initial pilot, printed questionnaires were sent to all junior trainees and educational supervisors in the training scheme in July 2007, and again in November 2007 (i.e. both before and after the introduction of WPBAs in August 2007). This allowed all junior psychiatrists to participate anonymously after at least 4 months in post, giving a potential total of 15 trainees and 15 supervisors in each data collection. Trainees and supervisors received identical questionnaires (except for appropriate grammatical changes), and each questionnaire was numbered to allow data from corresponding trainees and supervisors to be matched.

The content of the questionnaires was based on information found in the RCPsych guidance and findings from previous studies [1,8,9]. It was identical for each data collection, with the exception of the addition of a question about the extent of ES usage for WPBAs in the second cycle.

The actual and ideal frequency and duration of ES were established, including reasons for any deviation from 1 hour of ES weekly. Structure and content were ascertained. Opinions on usefulness and responsibility were sought using numerical scales (0 = "not useful" to 10 = "very useful", and 0 = "trainee responsible" to 10 = "supervisor responsible", respectively). The extent of usage of ES for WPBAs was also assessed. Participants were invited to make comments about ES at the end of each questionnaire. No changes were implemented between each data collection, other than the introduction of WPBAs.

All data was entered into Microsoft Excel worksheets and descriptive statistics used due to the nature of the information and sample size. The project was registered with the Clinical Effectiveness Department, who advised that it did not meet the criteria for requiring ethical approval (as this was an internal audit and survey of colleagues, not involving patients, and voluntary and anonymous for all participants).

## Results

11 trainees and 11 supervisors returned completed questionnaires in Data collection 1 (D1), giving a 73% response rate. Data collection 2 (D2) was similar, with 10 trainees and 10 supervisors participating (67%). Foundation Year 2 and General Practice trainees (junior doctors gaining brief experience in psychiatry as part of non-psychiatric training) participated alongside trainees pursuing a career in psychiatry. D1 was comprised of 11 paired responses, i.e. all completed questionnaires from trainees could be matched with questionnaires from their corresponding supervisors. D2 consisted of 6 matched pairs and 8 single questionnaires (from 4 trainees and 4 different supervisors).

### Frequency and duration

Data is presented as percentages for trainees [supervisors]. All trainees and supervisors reported that ES took place (Table 1). In D1, 54% [73%] indicated that ES happened at least 3 times monthly, compared to 60% [60%] in D2. 27% [18%] reported ES happening twice monthly in D1, compared to 40% [30%] in D2. 18% [9%] indicated ES taking place less than monthly in D1, compared to 0% [10%] in D2. The most common reasons given for not achieving weekly ES were annual/study leave, clinical commitments and sick leave for both trainees and supervisors.

**Table 1:** Frequency and duration of educational supervision

	Data collection 1* %		Data collection 2* %	
	Trainees	Consultants	Trainees	Consultants
	(11^†^)	(11^†^)	(10^†^)	(10^†^)
**Frequency**				
				
Weekly	36	64	50	20
3 times monthly	18	9	10	40
2 times monthly	27	18	40	30
Monthly	0	0	0	0
Less frequent	18	9	0	10
				
**Duration**				
				
>60 minutes	9	18	10	0
60	36	45	60	50
45–60	18	18	0	30
45	9	9	0	10
30–45	0	0	30	10
30	9	9	0	0
15–30	9	0	0	0
15	0	0	0	0
0–15	9	0	0	0

* 15 trainees and 15 supervisors invited to participate

† = number of participants

In D1, 64% [82%] indicated that ES lasted around 60 minutes, compared to 70% [80%] in D2. 36% [18%] reported ES lasting 45 minutes or less in D1, compared to 30% [20%] in D2. There was no clear correlation between frequency and duration of ES.

With regards to ideal frequency and duration of ES, 73% [82%] indicated it should take place at least 3 times monthly in D1, compared to 90% [90%] in D2. 64% [82%] opined that ES should last around 60 minutes in D1, compared to 70% [90%] in D2.

There were no consistent differences between trainees and supervisors. Supervisors were more likely to report more frequent ES in D1, with the converse in D2. There was generally good agreement between matched trainees and supervisors, with 1 significant exception in D1 (where the trainee reported ES as happening less than monthly, and their supervisor indicated it happened weekly). With regards to ES duration, several trainees reported significantly shorter sessions than their supervisors in D1, with the supervisors tending to indicate durations of between 15 to 30 minutes longer than their trainees. Where there was a discrepancy, there was also a trend for supervisors to consider ideal ES duration to be longer than their trainees.

### Structure and content

There was marked variability in the structure and content of ES reported by trainees and supervisors (Table 2). On average, neither trainees nor supervisors were consistently more likely to indicate the presence of different aspects of ES in either data collection. However, there was a trend for supervisors to report the setting of ground rules, management training, pastoral care, feedback on performance and supervision in writing reports more often than trainees.

**Table 2:** Structure and content of educational supervision

	Data collection 1* %		Data collection 2* %	
	Trainees	Supervisors	Trainees	Supervisors
	(11^†^)	(11^†^)	(10^†^)	(10^†^)
**Structure**				
				
Ground rules set	36	82	50	70
Expectations discussed	82	100	80	70
Prior training reviewed	64	82	80	80
Learning & training goals set	82	73	70	70
Educational plan written	55	27	50	40
Pre-set agenda for each session	18	36	20	10
Written record of each session	18	27	50	30
				
*Logbook reviewed regularly*^	*14*	*14*	*0*	*0*
*Logbook kept up-to-date*^	*57*	*14*	*50*	*0*
*Logbook useful*^	*14*	*0*	*0*	*0*
				
**Content**				
				
Clinical management	82	100	90	80
Evidence-based medicine	45	64	50	60
Teaching how to teach others	18	27	10	10
Research	55	36	20	40
Management training	18	55	10	30
Working with the multidisciplinary team	100	73	50	90
Pastoral care	36	73	40	70
Exam practice	18	55	40	20
Case presentations	55	73	40	50
Presenting at meetings	9	27	20	10
Setting learning objectives & priorities	64	73	60	50
Feedback on performance	73	100	50	90
Reviewing casenote entries & letters	64	82	40	50
Supervision in writing reports	0	36	10	40
Career guidance	73	73	70	40
Audit	55	45	60	50
Psychotherapy	18	18	10	10
Personal issues	45	55	40	30
Other	0	9^+^	0	0

* 15 trainees and 15 supervisors invited to participate

† = number of responses

^ *7 psychiatry trainees in Data collection 1, 6 in Data collection 2*

^+ ^Specific clinical topics

### Usefulness and responsibility

Both trainees and supervisors generally reported ES as being useful to some degree for a variety of purposes, and indicated that responsibility for ES should be shared equally between them (Table 3).

**Table 3:** Usefulness of and responsibility for educational supervision

	**Data collection 1**		**Data collection 2**	
	Trainees	Supervisors	Trainees	Supervisors
	**Median**			
	**(0 = "not useful" to 10 = "very useful")**			
				
**Usefulness**				
				
Career guidance	8	6	7	6
Management of clinical cases	8	7	8	8
Exam preparation	6	5	5	6
Education in general	7	7	7	7
Pastoral care	7	6	7	7
Performance feedback	8	8	8	8
Building a personal relationship	8	8	8	8
				
	**Median**			
	**(0 = "trainee responsible" to 10 = "supervisor responsible")**			
				
**Responsibility**	5	5	5	5

### Workplace-based assessments

60% of both trainees and supervisors reported using ES for WPBAs, with 40% [30%] indicating that 25% of ES time was used for this purpose, 10% [30%] indicating 50% usage, and 10% [0%] indicating 75% usage.

## Discussion

The response rate of 70% was in keeping with other surveys of ES [5,8,9]. Approximately 60% of both trainees and supervisors reported ES at least 3 times monthly, somewhat less than previous studies, and about 75% of ES sessions lasted around 60 minutes. This suggests that up to 40% of trainees only had ES twice per month at most, and around 25% received less than 1 full hour of ES – this clearly falls short of the RCPsych requirements.

Comments from trainees and supervisors revealed that annual/study leave, clinical commitments, lack of planning and perceptions that 1 hour of ES weekly is "irksome and never necessary" or possibly "an ideal – probably a luxury not attainable in the current NHS" may be contributing to shorter, less frequent ES sessions. Overall shorter working hours for trainees, with changing shift patterns which include nightshifts and compensatory rest, combined with shorter 4 month posts and a mixture of psychiatric and non-psychiatric trainees are all challenges in achieving "gold standard" ES, especially if neither trainees nor supervisors see it as a priority.

In addition to these opinions and practical challenges, supervisors' uncertainty over structure continues to be an issue. One supervisor commented that "We really don't have clear guidelines on what we are expected to do". While it is important that ES is flexible and tailored to serve individual trainees' unique professional and personal needs, there are certain components that are relevant for all trainees, and clear guidelines on structure and content have been suggested, assessed and published [7-9].

There were some discrepancies between what was reported by supervisors and trainees. One possible explanation for these may simply be recall bias, in that supervisors/trainees retrospectively remembered and reported the presence or absence of certain things that either pleased, disappointed or interested them. It may also be the case that trainees did not identify certain aspects of ES when their supervisors did not make them explicit, e.g. setting ground rules and giving feedback. This would fit with anecdotal evidence from the local medical school at the University of Glasgow, where it has been reported that medical students frequently fail to identify when their supervisors give them feedback unless it is either written down or presented explicitly to them as "feedback". In part because of small numbers, this study was not designed to identify any significant relationships between individuals' previous and current experience of ES and their opinions on ideal frequency or duration.

As the quality of supervision has been reported as being the single most important factor in determining trainee satisfaction, it is essential that trainees and supervisors alike make the most of this aspect of postgraduate psychiatric education and training [15]. All supervisors should ensure that they are aware of the published guidance and use it to adapt ES to each of their trainees' individual needs. Supervisors may also benefit from the recently developed RCPsych College Accredited Training Module in ES, where observed role-play is used to train supervisors in a variety of potential ES scenarios [16]. In addition, college tutors (identified psychiatrists with responsibility for overseeing postgraduate training and education in individual training schemes) have key roles to play in drawing supervisors' attention to the published guidance and training opportunities, and monitoring the quality of ES provided through feedback from trainees.

Although trainees and supervisors agree that responsibility for ES is shared equally between them, it may well be the case that each should take relatively more responsibility for different aspects. For example, perhaps supervisors should take the lead in structuring ES by ensuring that 1 hour of ES is prioritised and timetabled weekly, and by advising on content at the start of the trainees' posts. Conversely, trainees should take more responsibility for the content of ES as their attachments progress, particularly as the time belongs to them and should be tailored to their individual educational needs. The old adage of "the more you put in to it, the more you'll get out of it" is undoubtedly true for both supervisors and trainees, and it is significant to note that Lawley *et al*. (1995) found that the quality of supervision was subjectively improved by mutually agreeing agendas and preparing in advance [17]. Following the RCPsych guidance about using appropriate documentation such as that required for WPBAs is likely to facilitate the shared responsibility for ES [18].

WPBAs may well facilitate and enhance various aspects of ES, including its occurrence, frequency and duration. As a required component of approved postgraduate training curricula and assessment, WPBAs certainly provide motivation for trainees to meet regularly with their supervisors for the purpose of focusing on knowledge, skills and attitudes which are relevant for senior psychiatric practice [19]. Likewise, WPBAs may help to provide a degree of clarity for some of the structure and content of ES. If done well, and trainees ensure that they are assessed by different senior psychiatrists and others within the multidisciplinary team, there is the potential for WPBAs to improve and broaden psychiatrists' skills in a more reliable and consistent manner than previously achieved [11]. Hopefully this will benefit patients and colleagues alike.

The RCPsych is keen to encourage the development of mentoring relationships for all psychiatrists and mentoring is a specified element of the educational supervisor's role [2,18]. However, as one supervisor in this study commented, there can be "tension between mentoring and [the] objective appraisal of [a trainee's] performance". A conflict of interests between mentoring and assessment has been discussed in both the healthcare and educational literature [20,21]. Identified tensions include the "moral dilemma" of judging performance while being a mentor, and the finding that mentors' assessments are more favourable than those of non-mentors [21,22]. Concerningly, mentors have been reported as "failing to fail" their mentees even when they have doubts about their performance [23]. This in itself merits ensuring that trainees' WPBAs are not done solely by their supervisors. One further issue for psychiatry trainees is that using precious ES time for WPBAs introduces the potential for the increased emphasis on their appraisal to displace other educational needs. This is especially significant when ES as currently provided is falling short of existing RCPsych standards. It is therefore important that the use of ES time for WPBAs is monitored to ensure that ES remains tailored to individual trainees' needs. It is reassuring that this study in one typical UK basic psychiatry training scheme did not identify any negative effects of WPBAs on ES, but this should be subject to ongoing review across psychiatric training in the UK to ensure that trainees continue to receive high quality personalised ES which fulfils RCPsych requirements.

## Conclusion

ES continues to be an important component of psychiatric training. The recent introduction of WPBAs as a key part of the assessment of psychiatric trainees appears to be complementary to several of the aims of ES. However, using ES for WPBAs introduces the potential for tension between trainees' education and their assessment by emphasising certain training issues at the expense of others. The impact of reduced training time, WPBAs and uncertainties over ES structure and content should be monitored to ensure that its benefits are maximised by remaining tailored to individual trainees' needs. Educational supervisors should become familiar with the RCPsych and associated guidance on ES and perhaps avail themselves of specific training via the RCPsych. Trainees should take the initiative with both ES and WPBAs and share responsibility for them with their supervisors. College tutors should support supervisors by raising awareness of the published guidance and training opportunities, and by monitoring the provision and quality of ES.

This study does have several limitations, including relatively small numbers. However, the findings are consistent both with previous studies and anecdotal evidence, and the NHS Ayrshire and Arran scheme is fairly typical in size and structure to other rural training schemes in Scotland. Another limitation is the lack of data on specific aspects of WPBAs. For example, it would be useful to know exactly how trainees and supervisors are using ES for which WPBAs, and how different WPBAs serve the existing ends of ES. Future research should address these issues, and perhaps seek to compare practice in different training schemes. It is encouraging, however, that despite the time pressures on psychiatrists in the UK, the extra responsibilities of WPBAs and the uncertainties over structure and content, ES is still considered to be useful by trainees and supervisors alike.

## List of abbreviations used

ES: educational supervision; NHS: National Health Service; RCPsych: Royal College of Psychiatrists; UK: United Kingdom; WPBAs: Workplace-based assessments.

## Competing interests

The author declares that they have no competing interests.

## Authors' contributions

As the sole author, TEJ undertook all the work involved in this study, including the planning, literature review, designing the questionnaire, data collection and processing, and writing-up.

## Pre-publication history

The pre-publication history for this paper can be accessed here:



## References

[B1] Royal College of Psychiatrists (2003). Basic Specialist Training Handbook.

[B2] Royal College of Psychiatrists (2008). Postgraduate training in psychiatry: Essential information for trainees and trainers (Occasional Paper OP65).

[B3] Robertson JR, Dean A (1997). General professional training: consultant supervision of trainees. Advances in Psychiatric Treatment.

[B4] Royal College of Psychiatrists (1997). Organising Tutors' Induction Pack for the Personal Training File (Trainees' Log Book).

[B5] Herriot P, Bhui K, Lelliot P (1994). Supervision of trainees. Psychiatric Bulletin.

[B6] Azuonye IO (1997). Educational supervision sessions between consultants and trainees. Psychiatric Bulletin.

[B7] Cottrell D (1999). Supervision. Advances in Psychiatric Treatment.

[B8] Sembhi S, Livingston G (2000). What trainees and trainers think about supervision. Psychiatric Bulletin.

[B9] Ho H, McConville P (2004). Who's happy with supervision?. Psychiatric Bulletin.

[B10] Department of Health (2007). A Guide to Postgraduate Specialty Training in the UK: The Gold Guide.

[B11] Brown N, Doshi M (2006). Assessing professional and clinical competence: the way forward. Advances in Psychiatric Treatment.

[B12] Brown N, Bhugra D (2005). The European Working Time Directive. Psychiatric Bulletin.

[B13] Tsouroufli M, Payne H (2008). Consultant medical trainers, modernising medical careers (MMC) and the European time directive (EWTD): tensions and challenges in a changing medical education context. BMC Med Educ.

[B14] Carr S (2003). Education of senior house officers: current challenges. Postgrad Med J.

[B15] Faruqui RA, Ikkos G (2007). Poorly performing supervisors and trainers of trainee doctors. Psychiatric Bulletin.

[B16] Royal College of Psychiatrists (2007). Annual Review 2007.

[B17] Lawley D, Proudlove D, Bestley J (1995). Supervision of trainees. Psychiatric Bulletin.

[B18] Royal College of Psychiatrists (2008). Mentoring and Coaching (Occasional Paper OP66).

[B19] Postgraduate Medical Education and Training Board (2008). Standards for curricula and assessment systems.

[B20] Taherian K, Shekarchian M (2008). Mentoring for doctors. Do its benefits outweigh its disadvantages?. Med Teach.

[B21] Anforth P (1992). Mentors, not assessors. Nurse Educ Today.

[B22] Coulson CC, Kunselman AR, Cain J, Legro RS (2000). The mentor effect in student evaluation. Obstet Gynecol.

[B23] Rutkowski K (2007). Failure to fail: assessing nursing students' competence during practice placements. Nurs Stand.

